# Long-Term Follow-Up of JCOG1008, a Randomized Phase II/III Trial of Chemoradiotherapy Comparing 3-Weekly Cisplatin With Weekly Cisplatin in Postoperative Head and Neck Cancer

**DOI:** 10.1200/JCO-25-01708

**Published:** 2026-06-26

**Authors:** Makoto Tahara, Naomi Kiyota, Takeshi Kodaira, Hiroshi Nishino, Yukinori Asada, Hiroki Mitani, Yuji Hirayama, Yusuke Onozawa, Naoki Nishio, Nobuhiro Hanai, Akira Ohkoshi, Hiroki Hara, Nobuya Monden, Masato Nagaoka, Shujiro Minami, Kaoru Tanaka, Takashi Fujii, Seiichi Yoshimoto, Tsutomu Ueda, Yo Kishimoto, Akihiro Homma, Nobuhiko Oridate, Kenji Okami, Hirokazu Uemura, Katsunori Katagiri, Tomoko Yamazaki, Takahiro Asakage, Yuki Saito, Shigeyuki Murono, Junki Mizusawa, Kenichi Nakamura, Ryuichi Hayashi, on behalf of the Head and Neck Cancer Study Group of the Japan Clinical Oncology Group (JCOG-HNCSG), Akihiro Homma

**Affiliations:** ^1^ National Cancer Center Hospital East, Kashiwa, Japan; ^2^ Kobe University Hospital, Cancer Center, Kobe, Japan; ^3^ Aichi Cancer Center Hospital, Nagoya, Japan; ^4^ Jichi Medical University Hospital, Shimotsuke, Japan; ^5^ Miyagi Cancer Center, Natori, Japan; ^6^ Cancer Institute Hospital, Tokyo, Japan; ^7^ Hyogo Cancer Center, Akashi, Japan; ^8^ Shizuoka Cancer Center, Shizuoka, Japan; ^9^ Nagoya University Hospital, Nagoya, Japan; ^10^ Tohoku University Hospital, Sendai, Japan; ^11^ Saitama Cancer Center, Ina, Japan; ^12^ NHO Shikoku Cancer Center, Matsuyama, Japan; ^13^ Jikei University School of Medicine, Tokyo, Japan; ^14^ NHO Tokyo Medical Center, Tokyo, Japan; ^15^ Kindai University Hospital, Sakai, Japan; ^16^ Osaka International Cancer Institute, Osaka, Japan; ^17^ National Cancer Center Hospital, Tokyo, Japan; ^18^ Hiroshima University Hospital, Hiroshima, Japan; ^19^ Kyoto University, Kyoto, Japan; ^20^ Hokkaido University Hospital, Sapporo, Japan; ^21^ Yokohama City University Hospital, Yokohama, Japan; ^22^ Tokai University School of Medicine, Isehara, Japan; ^23^ Nara Medical University, Kashihara, Japan; ^24^ Iwate Medical University, Shiwa, Japan; ^25^ Saitama Medical University International Medical Center, Hidaka, Japan; ^26^ Institute of Science Tokyo Hospital, Tokyo, Japan; ^27^ The University of Tokyo Hospital, Tokyo, Japan; ^28^ Fukushima Medical University Hospital, Fukushima, Japan; ^29^ Japan Clinical Oncology Group Data Center/Operations Office, National Cancer Center Hospital, Tokyo, Japan

## Abstract

The interim analysis of JCOG1008 demonstrated that chemoradiotherapy with cisplatin 40 mg/m^2 ^once a week for seven cycles (weekly cisplatin) was noninferior in terms of overall survival (OS) compared with chemoradiotherapy with cisplatin 100 mg/m^2 ^once every 3 weeks for three cycles (3-weekly cisplatin) for postoperative high-risk locally advanced squamous cell carcinoma of the head and neck (LA-SCCHN). This report presents the long-term follow-up results of JCOG1008. In this phase II/III trial, patients with postoperative high-risk LA-SCCHN were randomly assigned to receive either chemoradiotherapy with 3-weekly cisplatin or with weekly cisplatin. A total of 261 patients were enrolled. At the final analysis, with a median follow-up of 5.6 years, 5-year OS was 58.7% in the 3-weekly arm and 71.2% in the weekly arm (hazard ratio, 0.76 [95% CI, 0.52 to 1.12 [<1.32]]), confirming the noninferiority of the weekly regimen. Both 5-year relapse-free survival (RFS) and 5-year local RFS were numerically better in the weekly arm. No late adverse event showed more than a 10% difference between the two arms. In conclusion, 5-year follow-up confirmed that chemoradiotherapy with weekly cisplatin is noninferior in OS to chemoradiotherapy with 3-weekly cisplatin in patients with postoperative high-risk LA-SCCHN. These results further support the use of weekly cisplatin plus radiotherapy as a reasonable alternative for this patient population.

## INTRODUCTION

Postoperative chemoradiotherapy with cisplatin 100 mg/m^2 ^once every 3 weeks for three cycles (3-weekly cisplatin) is standard for high-risk locally advanced squamous cell carcinoma of the head and neck (LA-SCCHN) but causes significant toxicity, which limits tolerability.^1-4^ Weekly cisplatin is often used because of its better safety,^5-12^ with meta-analyses showing comparable efficacy although randomized evidence remains limited.^13-18^

The JCOG1008 interim analysis showed noninferior overall survival (OS) and better acute safety with cisplatin 40 mg/m^2 ^once a week for seven cycles (weekly cisplatin) versus 3-weekly dosing.^19^ Here, we present the final 5-year results on long-term efficacy and late toxicity.

## PATIENTS AND METHODS

### Study Design and End Points

JCOG1008 (the Japan Registry of Clinical Trials, Number: jRCTs031180135) is a multi-institutional, open-label, randomized, noninferiority phase II/III trial in Japan.^19^ Eligible patients were randomly assigned 1:1 to receive chemoradiotherapy with either 3-weekly or weekly cisplatin, using minimization with random adjustment for institution and high-risk factors.

The primary end point was OS; secondary end points included relapse-free survival (RFS), local RFS, nutrition support–free survival, nonhospitalized treatment duration, and adverse events.

### Statistical Analysis

The primary analysis was conducted in the intention-to-treat (ITT) population, with a prespecified per-protocol (PP) analysis excluding patients who did not receive the protocol treatment.

OS was analyzed using a stratified Cox model with treatment as a covariate and stratification by relapse risk factors (margin positivity and extracapsular extension); this constituted the primary analysis. A secondary analysis additionally adjusted for T stage, N stage, and primary site.

The noninferiority margin (hazard ratio [HR], 1.32) was set to preserve at least 30% of cisplatin’s established benefit over radiotherapy (RT) alone, on the basis of a previous meta-analysis.^8^

At the third interim analysis, noninferiority of weekly cisplatin for OS was confirmed, and the JCOG Data and Safety Monitoring Committee recommended early trial termination. Follow-up continued, and the final analysis was conducted 5 years after accrual completion. Additional details are provided in the Data Supplement (online only).

## RESULTS

### Patient Characteristics

A total of 261 patients were randomized (132 in the 3-weekly arm, 129 in the weekly arm). Baseline characteristics were generally balanced, except for more T4 (68 *v* 59), N2-N3 (112 *v* 106), and hypopharyngeal patient cases (45 *v* 37) in the 3-weekly arm and more oropharyngeal patient cases in the weekly arm (45 *v* 37; Table 1). Treatment compliance is shown in the Data Supplement (Table S1). Estimated dose intensity was higher in the weekly arm (33.6 *v* 29.3 mg/m^2^ once a week).

**TABLE 1.** Baseline Characteristics

Characteristic	3-Weekly Cisplatin (n = 132)	Weekly Cisplatin (n = 129)	Total (N = 261)
Age, years, median (IQR)	62 (55-68)	61 (53-66)	62 (54-67)
Sex			
Men	110 (83)	110 (85)	220 (84)
Women	22 (17)	19 (15)	41 (16)
ECOG performance status			
0	92 (70)	93 (72)	185 (71)
1	40 (30)	36 (28)	76 (29)
Primary site			
Oral cavity	61 (46)	60 (46)	121 (46)
Larynx	12 (9)	11 (9)	23 (9)
Hypopharynx	45 (34)	37 (29)	82 (31)
Oropharynx	14 (11)	21 (16)	35 (14)
p16-positive	12 (9)	15 (12)	27 (10)
p16-negative	2 (2)	5 (4)	7 (3)
Unknown	0	1 (1)	1 (0.4)
High-risk factors			
Positive margin	43 (33)	42 (33)	85 (33)
Extranodal extension	112 (85)	109 (85)	221 (85)
Pathologic T stage			
T1	13 (10)	7 (5)	20 (8)
T2	26 (20)	40 (31)	66 (25)
T3	25 (19)	23 (18)	48 (18)
T4	68 (51)	59 (46)	127 (49)
Pathologic N stage			
N0	9 (7)	6 (5)	15 (5)
N1	10 (7)	15 (12)	25 (10)
N2	107 (81)	104 (81)	211 (81)
N3	5 (4)	2 (1)	7 (3)
Nx	1 (1)	2 (1)	3 (1)
Pathologic stage			
III	9 (7)	11 (9)	20 (8)
IVA	117 (88)	113 (88)	230 (88)
IVB	5 (4)	3 (2)	8 (3)
Unknown	1 (1)	2 (1)	3 (1)

NOTE. Data are No. (%) unless otherwise specified.

Abbreviation: ECOG, Eastern Cooperative Oncology Group.

### Efficacy

**FIG 1.**
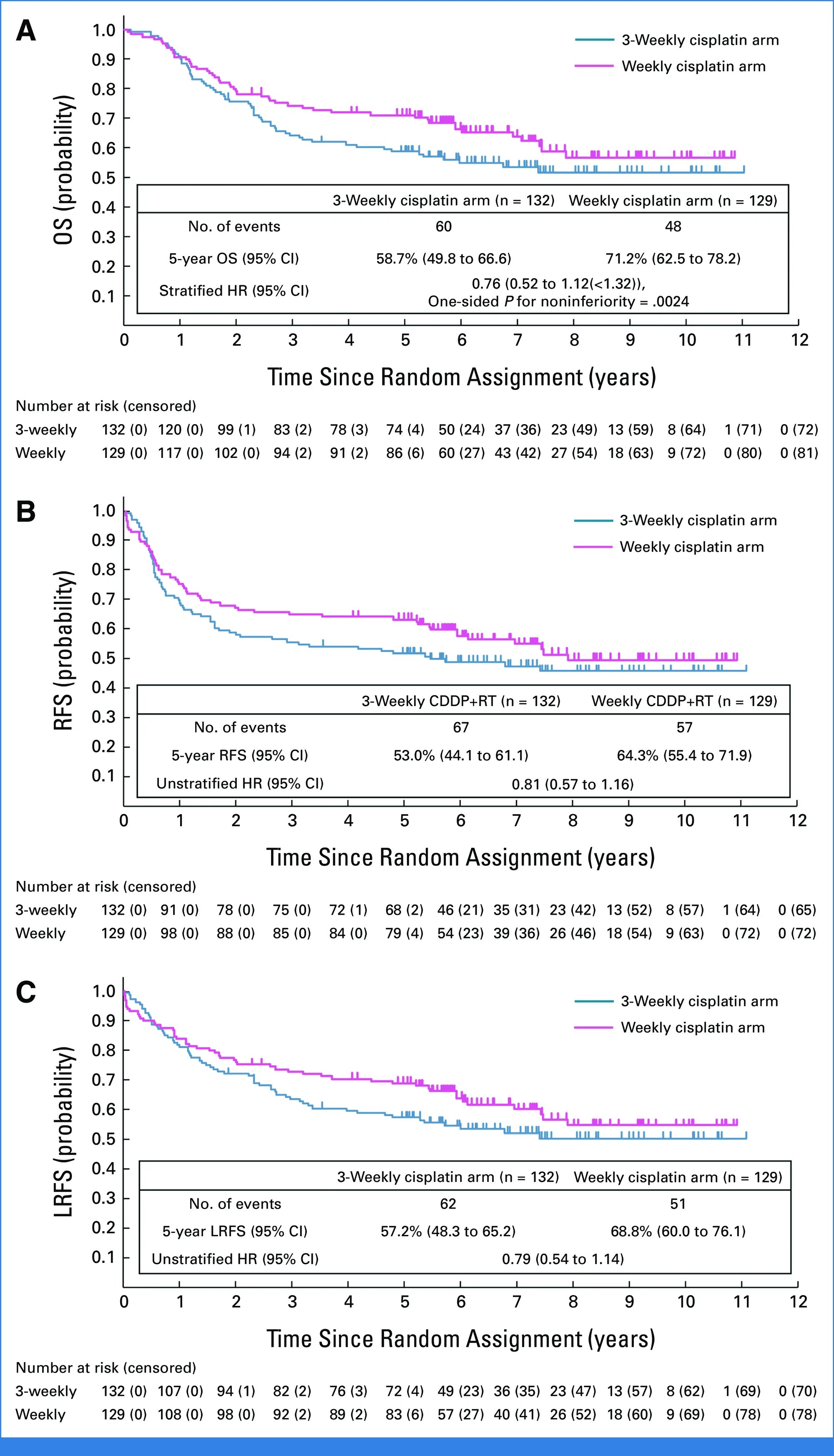
(A) Kaplan-Meier curve for OS for all randomly assigned patients. The symbols indicate censored observations. HRs were computed using a stratified Cox proportional hazards model, and the *P* values were from a stratified log-rank test. (B) Kaplan-Meier curve for RFS for all randomly assigned patients. (C) Kaplan-Meier curve for LRFS for all randomly assigned patients. HRs were estimated using a stratified Cox proportional hazards model, with stratification by high-risk factors for relapse (microscopic resection margin positivity and extracapsular nodal extension). HR, hazard ratio; LRFS, local relapse-free survival; OS, overall survival; RFS, relapse-free survival; RT, radiotherapy.

On final analysis (median follow-up, 5.6 years), 5-year OS was 58.7% in the 3-weekly arm versus 71.2% in the weekly arm (HR, 0.76 [95% CI, 0.52 to 1.12 [<1.32]]), confirming noninferiority (Fig 1A). The adjusted HR was 0.88 (95% CI, 0.60 to 1.29), and the PP analysis yielded an HR of 0.75 (95% CI, 0.51 to 1.12; Data Supplement, Table S2 and Fig S1).

**FIG 2.**
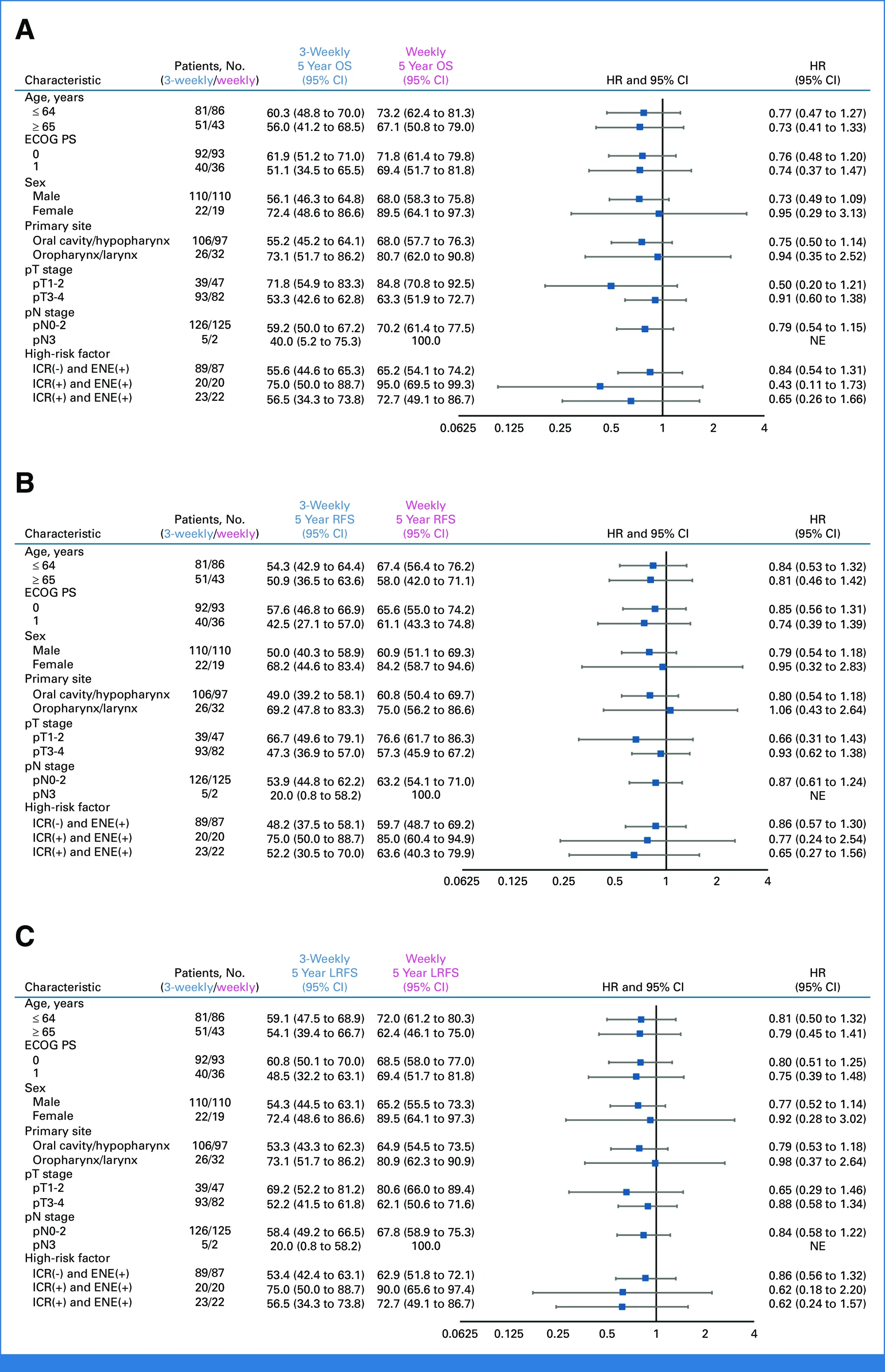
(A) The plot of unstratified HRs for death in the analysis of treatment effect according to baseline demographic and clinical subgroups. (B) The plot of unstratified hazard ratios for death or recurrence in the analysis of treatment effect according to baseline demographic and clinical subgroups. (C) The plot of unstratified hazard ratios for death or local recurrence in the analysis of treatment effect according to baseline demographic and clinical subgroups. Subsites were grouped a priori into oropharynx/larynx and oral cavity/hypopharynx to reflect broadly similar survival tiers reported in population datasets and to avoid underpowering of strata; see the Discussion for references. ECOG PS, Eastern Cooperative Oncology Group performance status; ENE, extranodal extension; HPX, hypopharynx; HR, hazard ratio; ICR, incomplete resection; LRFS, local relapse-free survival; LX, larynx; NE, not evaluable; OC, oral cavity; OPX, oropharynx; RFS, relapse-free survival.

Five-year RFS was 53.0% in the 3-weekly arm versus 64.3% in the weekly arm (HR, 0.81 [95% CI, 0.57 to 1.16]), and local RFS was 57.2% versus 68.8% (HR, 0.79 [95% CI, 0.54 to 1.14]; Figs 1B and 1C). Distant metastases (34 [25.8%] *v* 24 [18.6%]) and postrecurrence therapy (47 [36.4%] *v* 37 [28.7%]) were numerically more frequent in the 3-weekly arm (Data Supplement, Tables S3 and S4). Subgroup analyses consistently favored weekly cisplatin (Fig 2).

### Safety

As previously noted, the weekly arm showed better acute safety.^19^ Two treatment-related deaths occurred in the weekly arm (laryngeal edema and myelosuppression) and none in the 3-weekly arm (Data Supplement, Table S2). No late toxicity differed by ≥10% between the arms (Data Supplement, Table S5). Treatment compliance details are in the Data Supplement (Table S1).

Five-year nutrition support–free survival was 54.3% versus 63.9% (feeding tube use: gastrostomy—3-weekly, n = 3; weekly, n = 2; nasogastric—weekly, n = 2; Data Supplement, Table S8). Because weekly cisplatin is rarely given in outpatient settings in Japan, nonhospitalized treatment duration was similar between the arms (Data Supplement, Table S6).

Radiation protocol violations occurred in three patients per arm, evenly distributed between 3D-CRT and IMRT, and were unlikely to affect outcomes (Data Supplement, Table S7).

## DISCUSSION

This final 5-year analysis confirms that weekly cisplatin treatment is noninferior in terms of OS. The ITT and PP analyses showed consistent results, confirming robustness. RT deviations occurred in three patients per arm, with no crossover, indicating minimal impact on outcomes.

Additionally, the weekly cisplatin arm showed numerically better clinical outcomes, including OS, RFS, and local RFS, compared with the 3-weekly arm.

Better outcomes in the weekly arm may relate to higher estimated cisplatin dose intensity (33.6 *v* 29.3 mg/m^2^ once a week),^19^ which may also explain discrepancies with an Indian phase III trial using 30 mg/m^2^ once a week.^14^ However, because cisplatin timing differed between the arms, dose intensity could only be estimated, and its relationship with prognosis remains complex, likely reflecting better tolerability rather than biologic superiority.

Weekly dosing may provide more consistent radiosensitization across RT fractions, whereas in the 3-weekly arm, hematologic toxicity sometimes delayed cisplatin administration until or after RT completion, when radiosensitization is likely reduced, despite a higher cumulative cisplatin dose.

More patients in the 3-weekly arm developed distant metastases, while 5-year local RFS was higher in the weekly arm. Improved local control with weekly cisplatin, as reported previously,^20-22^ may have contributed to better RFS and OS.

No late adverse event differed by more than 10% between the arms, suggesting limited impact of cisplatin dose or schedule on late toxicity. Severe late toxicities were less frequent than in earlier RTOG studies,^23^ possibly because of the shorter follow-up in the present study.

This study has several limitations. Random assignment was stratified by institution and key pathologic risk factors, yet baseline imbalances in T stage, N stage, and primary site could have influenced outcomes. Adjusted analyses yielded an HR of 0.88 (95% CI, 0.60 to 1.29), indicating that the noninferiority conclusion remained robust even after accounting for these imbalances, although they may have partially contributed to the numerically favorable results observed in the weekly arm. Therefore, future trials should include additional stratification factors to avoid such bias. The relatively wide noninferiority margin (HR, 1.32), although justified on the basis of historical effect preservation and feasibility considerations, remains a limitation of the study, and narrower margins should be considered in future trials.

Two treatment-related deaths occurred in the weekly arm, likely related to patient susceptibility or late RT toxicity rather than cisplatin itself. Late toxicities such as dysphagia, xerostomia, renal impairment, and hearing loss were also detailed.

The low proportion of oropharyngeal and human papillomavirus (HPV)–positive disease in our study is consistent with other postoperative trials, including KEYNOTE-689,^24^ and reflects the typical characteristics of postoperative populations rather than epidemiologic differences, as HPV-positive oropharyngeal cancer is not uncommon in Japan. Moreover, even in Western countries, many patients with HPV-positive oropharyngeal cancer undergo definitive chemoradiotherapy because of its high sensitivity, resulting in their underrepresentation in surgical and postoperative chemoradiotherapy trials.

Although limited to Japanese patients, weekly cisplatin is feasible given daily RT visits, and treatment practices align globally, supporting generalizability.

Although quality-of-life (QOL) data were not prospectively collected, late toxicity profiles related to airway and swallowing function, as well as surrogate functional outcomes such as nutrition support–free survival and feeding tube dependence, did not differ meaningfully between the two arms, suggesting no major between-group differences in patient-relevant functional outcomes. Cost-effectiveness analyses were not performed because both arms used cisplatin with similar clinical demands; future studies should include standardized QOL measures.

A further limitation is that patients ineligible for high-dose cisplatin were excluded; thus, the efficacy of weekly cisplatin in this population cannot be determined from our trial. Future studies focusing on high-dose–ineligible patients are required.

Finally, the trial began in 2012, and few patients received immunotherapy at recurrence, limiting extrapolation to current treatment contexts.

Several supplementary analyses from this study have been published.^25-27^ A biomarker study aimed at predicting clinical outcomes of postoperative chemoradiotherapy in high-risk LA-SCCHN is currently ongoing.

In conclusion, this 5-year analysis confirms the noninferiority of weekly cisplatin for OS, with favorable acute and comparable late toxicity versus the 3-weekly regimen. Weekly cisplatin plus RT represents a reasonable alternative for postoperative high-risk LA-SCCHN, although further studies are needed before universal adoption.

## Data Availability

A data sharing statement provided by the authors is available with this article at DOI https://doi.org/10.1200/JCO-25-01708. Anonymized individual participant data that underlie the results reported in this article will be shared after deidentification to investigators whose proposed data use has been approved by investigators of the JCOG Head and Neck Cancer Study Group identified for that purpose. Proposals should be directed to matahara@east.ncc.go.jp.

## APPENDIX

**TABLE A1.** Study Institutions

Implementing Medical Institution	Department	Principal Investigator
Hokkaido University Hospital	Otorhinolaryngology	Akihiro Homma
Iwate Medical University	Head and Neck Surgery	Kiyoto Shiga
Tohoku University Hospital	Otorhinolaryngology, Head and Neck Surgery	Takenori Ogawa
Miyagi Cancer Center	Head and Neck Medical Oncology	Tomoko Yamazaki
Fukushima Medical University Hospital	Otorhinolaryngology, Head and Neck Surgery	Shigeyuki Murono
Jichi Medical University	Otorhinolaryngology	Hiroshi Nishino
Saitama Cancer Center	Gastroenterology	Hiroki Hara
Saitama Medical University International Medical Center	Head and Neck Oncology, Otorhinolaryngology	Masashi Sugasawa
National Cancer Center Hospital East	Head and Neck Medical Oncology	Makoto Tahara
National Cancer Center Hospital	Head and Neck Surgery	Seiichi Yoshimoto
National Hospital Organization Tokyo Medical Center	Otorhinolaryngology	Shujiro Minami
Tokyo Medical and Dental University Hospital	Head and Neck Surgery	Takahiro Asakage
Jikei University Hospital	Otorhinolaryngology	Eiji Shimura
Cancer Institute Hospital of Japanese Foundation for Cancer Research	Head and Neck Surgery	Hiroki Mitani
The University of Tokyo Hospital	Otorhinolaryngology, Head and Neck Surgery	Mizuo Ando
Yokohama City University Hospital	Otorhinolaryngology	Nobuhiko Oridate
Tokai University Hospital	Otorhinolaryngology	Kenji Okami
Shizuoka Cancer Center	Unknown Primary	Yusuke Onozawa
Aichi Cancer Center	Head and Neck Surgery	Nobuhiro Hanai
Nagoya University Hospital	Otorhinolaryngology	Yasushi Fujimoto
Kyoto University Hospital	Otorhinolaryngology, Head and Neck Surgery	Koichi Omori
Kindai University Hospital	Oncology	Kazuhiko Nakagawa
Osaka International Cancer Institute	Head and Neck Surgery	Takashi Fujii
Kobe University Hospital	Cancer Center	Naomi Kiyota
Hyogo Cancer Center	Head and Neck Surgery	Shigemichi Iwae
Nara Medical University	Otorhinolaryngology, Head and Neck Surgery	Ichiro Ota
Hiroshima University Hospital	Otorhinolaryngology, Head and Neck Surgery	Tsutomu Ueda
National Hospital Organization Shikoku Cancer Center	Head and Neck, Thyroid Oncology	Nobuya Monden

NOTE. Fifty-eight in total (at the initiation of the study).
