# Supplementary material for: Long-Term Follow-Up of JCOG1008, a Randomized Phase II/III Trial of Chemoradiotherapy Comparing 3-Weekly Cisplatin With Weekly Cisplatin in Postoperative Head and Neck Cancer
Source: J Clin Oncol. 2026 Jun 26;44(27):2574–81. doi: 10.1200/JCO-25-01708 (PMC13581295; doi:10.1200/JCO-25-01708)
Supplement: Supplementary file 1 [file jco-44-2574-s001.pdf]

Supplementary Appendix

Supplementary online content for Makoto Tahara, Naomi Kiyota, et al for the appendix of 'Long-term Follow-up of JCOG1008, a Randomized Phase II/III Trial of Chemoradiotherapy Comparing 3-weekly Cisplatin with Weekly Cisplatin in Post-operative Head and Neck Cancer.

Table of Contents

Table S1. Treatment delivery, compliance with radiation therapy and chemotherapy

Table S2. Reason for death at 5-year follow-up

Table S3. Initial recurrence pattern at 5-year follow-up

Table S4. Subsequent therapy following recurrence

Table S5. Late toxicity at 5-year follow-up

Table S6. Treatment period for non-hospitalized patients during the permissible treatment period

Table S7. Quality assurance of RT

Table S8. Nutrition support–free survival

Figure S1. Overall survival in the per-protocol (PP) analysis at 5-year follow-up

Methodology for statistical analysis

In this final analysis, no multiplicity adjustment was applied, no hypothesis testing was performed, and no alpha was allocated because primary end points were met at previous analyses. All analyses were performed using SAS version 9.4. Missing data were censored at the time of last follow-up.

Because cisplatin was limited to the RT period in the weekly arm but allowed for up to 2 weeks post-RT in the 3-weekly arm, treatment duration differed and precise dose intensity calculation was not feasible. Therefore, dose intensity was estimated as the planned weekly dose (40 mg/m²/week in the weekly arm and 33.3 mg/m²/week in the 3-weekly arm) multiplied by the ratio of the mean actual cumulative dose to the planned cumulative dose (280 mg/m² for weekly and 300 mg/m² for 3-weekly).

Table S1. Treatment delivery, compliance with radiation therapy and chemotherapy

		No. of patients	
Parameter		3-weekly cisplatin (N=132)	Weekly cisplatin (N=129)
Completion of protocol treatment		123 (93.2%)	115 (89.1%)
Protocol treatment failure		1 (0.8%)	3 (2.3%)
Discontinuation due to AEs		1 (0.8%)	1 (0.8%)
Discontinuation due to patient refusal[#]		3 (2.3%)	0
Death during protocol treatment		0 (0.0%)	1 (0.8%)
Total RT dose (Gy)	Median (IQR)	66 (66-66)	66 (66-66)
Duration of RT (days)	Median (IQR)	49 (47-51)	49 (46-50)
Interval from surgery to RT initiation (days)	Median (IQR)	49 (42-56)	50 (43-56)
Cycles of cisplatin	Median (IQR)	3 (3-3)	6 (5-7)
Total cycle of cisplatin	0	3 (2.3%)	7 (5.4%)
	1	3 (2.3%)	0
	2	17 (12.9%)	3 (2.3%)
	3	109 (82.6%)	1 (0.8%)
	4	-	6 (4.7%)
	5	-	21 (16.3%)
	6	-	30(23.3%)
	7	-	61 (47.3%)
Dose reduction	no	118 (89.4%)	115 (89.1%)
	yes	11 (8.3%)	7 (5.4%)
Not receiving scheduled cisplatin per cycle			
Cycle 1	Delay	0	0
	Discontinuation	0	0
	Dose reduction	0	0
	Delay + Dose reduction	0	0
Cycle 2	Delay	24 (18.2%)	5 (3.9%)
	Discontinuation	2 (1.5%)	0

	Dose reduction	10 (7.6%)	4 (3.1%)
	Delay + Dose reduction	22 (16.7%)	0
Cycle 3	Delay	29 (22.0%)	6 (4.7%)
	Discontinuation	15 (11.4%)	0
	Dose reduction	10 (7.6%)	3 (2.3%)
	Delay + Dose reduction	11 (8.3%)	3 (2.3%)
Cycle 4	Delay	-	8 (6.2%)
	Discontinuation	-	1 (0.8%)
	Dose reduction	-	4 (3.1%)
	Delay + Dose reduction	-	0
Cycle 5	Delay	-	15 (11.6%)
	Discontinuation	-	5 (3.9%)
	Dose reduction	-	2 (1.6%)
	Delay + Dose reduction	-	6 (4.7%)
Cycle 6	Delay	-	19 (14.7%)
	Discontinuation	-	19 (14.7%)
	Dose reduction	-	5 (3.9%)
	Delay + Dose reduction	-	2 (4.7%)
Cycle 7	Delay	-	10 (7.8%)
	Discontinuation	-	24 (18.6%)
	Dose reduction	-	3 (2.3%)
	Delay + Dose reduction	-	0
Cumulative dose of cisplatin (mg/m^2)	Median (IQR)	280 (250-299)	239 (199-277)
Estimated dose intensity of cisplatin (mg/m^2)*	Median	29.3	33.6
Unscheduled RT interruptions	none	12 (9.1%)	14 (10.9%)
	yes	117 (88.6%)	108 (83.7%)
Reasons for RT interruption (multiple allowed)	Adverse events	29 (22.0%)	15 (11.6%)
	National holidays (excluding weekends)	105 (79.5%)	98 (76.0%)

	Machine failure / maintenance	29 (22.0%)	34 (26.4%)
	Patient preference	2 (1.5%)	5 (3.9%)
	Other	8 (6.1%)	6 (4.7%)
Total days of unscheduled RT interruption due to adverse events	Median (min-max)	3 (1-9)	3 (1-14)
Total days of unscheduled RT interruption due to non-adverse events	Median (min-max)	2 (1-7)	2 (1-9)
Cisplatin administered after RT completion		29 (22.0%)	0

[#] Related to AEs, IQR: interquartile range, Level 1: 100 mg/m^2 in 3 weekly arm, 40 mg/m^2 in weekly arm, Level 0: 80 mg/m^2 in 3 weekly arm, 30 mg/m^2 in weekly arm, Level -1: 60 mg/m^2 in 3 weekly arm, 20 mg/m^2 in weekly arm. *Calculated as planned weekly dose × (mean actual cumulative dose/planned cumulative dose)

Table S2. Reason for death at 5-year follow-up

Reason	3-weekly CDDP+RT (N=132)	Weekly CDDP+RT (N=129)
Recurrence	45 (34.1%)	32 (24.8%)
Other disease	10 (7.6%)	10 (7.8%)
Treatment-related death	0	2* (1.6%)
Unknown	5 (3.8%)	4 (3.1%)

*On day 14 after starting study treatment, one patient developed grade 4 neutropenia. This was followed by fever and diarrhea on day 16, progressive disseminated intravascular coagulation (DIC), and death on day 17.

*On day 327 after starting study treatment, another patient was found in cardiac arrest at home and passed away despite resuscitation efforts. Based on CT findings, the cause of death was determined to be asphyxiation due to laryngeal edema.

Table S3. Initial recurrence pattern at 5-year follow-up

Recurrent site	3-weekly cisplatin (N=55)	Weekly cisplatin (N=43)
All recurrence	55 (41.7%)	43 (33.3%)
Locoregional recurrence	16 (12.1%)	17 (13.2%)
Distant metastasis	34 (25.8%)	24 (18.6%)
Both	5 (3.8%)	2 (1.6%)

Table S4. Subsequent therapy following recurrence

Subsequent therapy	3-weekly cisplatin (N=132)	Weekly cisplatin (N=129)
Initial subsequent therapy following recurrence	47 (35.6%)	37 (28.7%)
Reradiation for locoregional recurrence	0	1 (0.8%)
Reradiation plus chemotherapy for locoregional recurrence	2 (1.5%)	4 (3.1%)
Systemic therapy for distant metastasis	31 (23.5%)	20 (15.5%)
5-FU + cisplatin + docetaxel	0	1 (0.8%)
Cetuximab + 5-FU + cisplatin	4 (3.0%)	5 (3.9%)
Cetuximab + 5-FU + certain drug*	4 (3.0%)	2 (1.6%)
Cetuximab + certain drug*	9 (6.8%)	2 (1.6%)
Docetaxel	3 (2.3%)	1 (0.8%)
S-1	3 (2.3%)	2 (1.6%)
Other systemic therapy	8 (6.1%)	7 (5.4%)
2nd line subsequent therapy following recurrence	32 (24.2%)	27 (20.9%)
Immune checkpoint inhibitor as subsequent therapy	7 (5.3%)	6 (4.7%)

* Available cytotoxic drug for head and neck cancer in Japan

- 85.5% (47/55) of patients in the 3-weekly arm and 86% (37/43) in the weekly arm received subsequent therapy following recurrence.
- The reasons for not receiving post-recurrence therapy are unknown because these data were not collected.

Table S5. Late toxicity at 5-year follow-up in ≥1% of patients in the safety subset (N=250)

Adverse reaction	3-weekly cisplatin (N=129)		Weekly cisplatin (N=121)	
	Any grade (%)	G3-4 (%)	Any grade (%)	G3-4 (%)
Any	118 (91.5)	13 (10.1)	114 (94.2)	16 (13.2)
Dry mouth	78 (60.5)	0	78 (64.5)	0
Dysgeusia	76 (58.9)	-	75 (62.0)	-
Dysphagia	52 (40.3)	6 (4.7)	56 (46.3)	6 (5.0)
Hypothyroidism	40 (31.0)	0	49 (40.5)	0
Neck edema	32 (24.8)	0	26 (21.5)	2 (1.7)
Hearing impairment	21 (16.3)	0	17 (14.0)	3 (2.5)
Tinnitus	21 (16.3)	0	8 (6.6)	0
Fatigue	15 (11.6)	0	22 (18.2)	0
Laryngeal edema	14 (10.9)	0	11 (9.1)	1 (0.8)
Peripheral sensory neuropathy	14 (10.9)	0	22 (18.2)	0
Osteonecrosis of jaw	9 (7.0)	3 (2.3)	4 (3.3)	0
Trismus	5 (3.9)	0	10 (8.3)	1 (0.8)
Lung infection	5 (3.9)	2 (1.6)	7 (5.8)	5 (4.1)
Dental caries	5 (3.9)	1 (0.8)	6 (5.0)	0
Peripheral motor neuropathy	4 (3.1)	0	4 (3.3)	0
Neck soft tissue necrosis	2 (1.6)	1 (0.8)	4 (3.3)	2 (1.7)

Table S6. Treatment period for non-hospitalized patients during the permissible treatment period*

	Median treatment period for non-hospitalized patients (range)	
Treatment arm	All registered patients (N=261)	All treated patients (N=251)
3-weekly arm	4 days (0-60)	4 days (0-60)
Weekly arm	9 days (0-64)	10 days (0-64)
	p-value=0.1156	*p*-value=0.0522

*The scheduled treatment period was 66 days.

Table S7. Quality assurance of RT

Quality Assurance (QA) results	3-weekly cisplatin<br>N=132<br>(N=129 for pts receiving CRT)	Weekly cisplatin<br>N=129<br>(N=122 for pts receiving CRT)
QA for RT		
Compliance	86 (65.2%)	86 (66.7%)
Deviation	34 (25.8%)	27 (20.9%)
Violation	3 (2.3%)	3 (2.3%)
Not assessable	1 (0.8%)	1 (0.8%)
Outside judgment	8 (5)	12 (5)
QA for 3D-RT	N=43	N=44
Compliance	19 (44.2%)	22 (50.0%)
Deviation	18 (41.9%)	18 (40.9%)
Violation	2 (4.7%)	1 (2.3%)
Not assessable	1 (2.3%)	1 (2.3%)
Outside judgment	3 (7.0%)	2 (4.5%)
QA for IMRT	N=86	N=78
Compliance	67 (77.9%)	64 (82.1%)
Deviation	16 (18.6%)	9 (11.5%)
Violation	1 (1.2%)	2 (2.6%)
Not assessable	0	0
Outside judgment	2 (2.3%)	3 (3.8%)

pts: patients

Definition of category

Compliance: A category where the protocol rules are fully adhered to.

Deviation: A category where there is a departure from protocol rules, but the impact on treatment outcomes is minimal.

Violation: A category where there is a departure from protocol rules, and the impact on treatment outcomes is significant.

Details of violations in patients treated with 3D-CRT and IMRT

3D-CRT:

- One patient was judged off-protocol because the prescribed dose exceeded the protocol allowance, with a single-fraction overdose of 20%; treatment was therefore terminated at 64 Gy.
- One patient's high-dose target volume did not adequately include the intended region.

- One patient’s normal organ dose constraints were not met.

IMRT:

- One patient’s dose coverage (D95) to the target volume was slightly below the protocol threshold, achieving 96.8% instead of the required 98%.
- One patient’s elective prophylactic region did not adequately include the specified area.
- One patient’s high-dose target volume did not fully include the intended region.

Table S8. Nutrition support–free survival

**3-weekly cisplatin (N=132)**	No. of patients							
	Baseline	2 months	6 months	12 months	24 months	38 months	48 months	60 months
Primary nutritional support method								
Oral intake	112	71	106	98	95	79	75	70
Gastrostomy	14	46	18	17	2	1	1	3
Nasogastric tube	3	12	2	1	1	1	1	0
Parenteral nutrition	0	0	0	2	0	1	0	0
Other	0	0	1	1	1	1	1	0
Missing	0	0	2	10	30	46	51	56
Nutrition support–free survival (Oral intake)								
Nutrition support–free survival	86.3%	55.0%	82.2%	76.0%	73.6%	61.2%	58.1%	54.3%
95%CI	79.7-92.1	46.0-63.8	74.5-88.4	67.7-83.1	65.2-81.0	52.3-69.7	49.1-66.8	45.3-63.1

**Weekly cisplatin (N=129)**	No. of patients							
	Baseline	2 months	6 months	12 months	24 months	38 months	48 months	60 months
Primary nutritional support method								
Oral intake	116	82	106	107	95	87	84	78
Gastrostomy	6	31	11	5	2	1	2	2
Nasogastric tube	0	7	2	1	1	0	0	2
Parenteral nutrition	0	1	0	0	0	0	0	0
Other	0	0	0	0	0	0	1	0
Missing	0	1	3	9	24	34	36	39
Nutrition support–free survival (Oral intake)								
Nutrition support–free survival	95.1%	67.2%	86.9%	87.7%	77.9%	71.3%	68.9%	63.9%
95%CI	89.6-98.2	58.1-75.4	79.6-92.3	80.5-93.0	69.5-84.9	62.4-79.1	59.8-76.9	54.8-72.4

Figure S1. Overall survival in the per-protocol (PP) analysis at 5-year follow-up

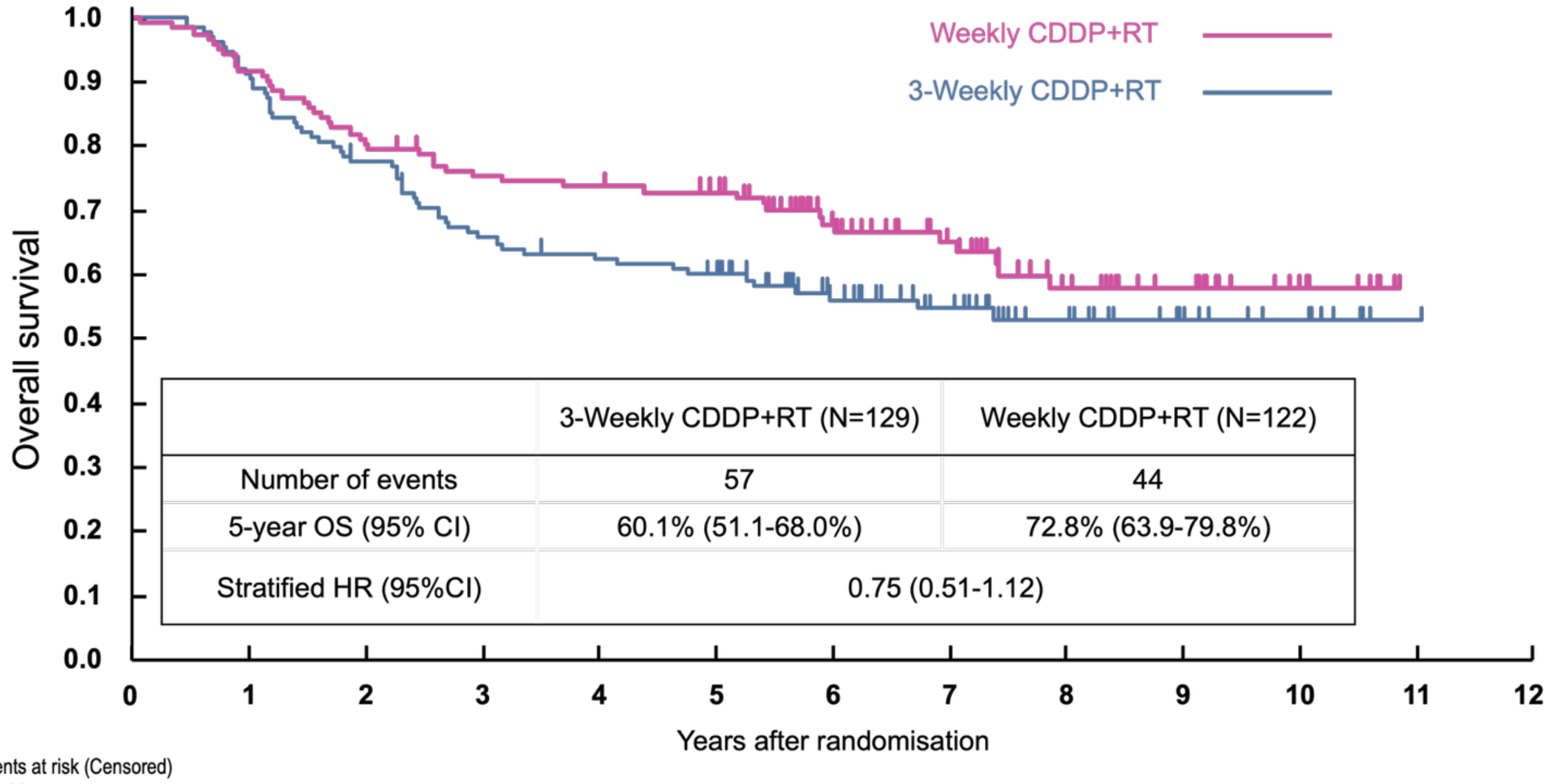

	3-Weekly CDDP+RT (N=129)	Weekly CDDP+RT (N=122)
Number of events	57	44
5-year OS (95% CI)	60.1% (51.1-68.0%)	72.8% (63.9-79.8%)
Stratified HR (95%CI)	0.75 (0.51-1.12)	

Patients at risk (Censored)

	0	1	2	3	4	5	6	7	8	9	10	11	12
3-weekly	129(0)	118(0)	99(1)	83(2)	78(3)	74(4)	50(24)	37(36)	23(49)	13(59)	8(64)	1(71)	0(72)
Weekly	122(0)	112(0)	98(0)	90(2)	88(2)	84(5)	59(25)	42(40)	26(52)	18(60)	9(69)	0(78)	0(78)
