# Supplementary material for: Long-Term Follow-Up of JCOG1008, a Randomized Phase II/III Trial of Chemoradiotherapy Comparing 3-Weekly Cisplatin With Weekly Cisplatin in Postoperative Head and Neck Cancer
Source: J Clin Oncol. 2026 Jun 26;44(27):2574–81. doi: 10.1200/JCO-25-01708 (PMC13581295; doi:10.1200/JCO-25-01708)
Supplement: Supplementary file 2 [file jco-44-2574-s002.pdf]

ASCO Journals Data Sharing Statement Questionnaire

**1. Manuscript Title**	Long-term Follow-up of JCOG1008, a Randomized Phase II/III Trial of Chemoradiot
**2. First Author Last Name**	Makoto Tahara
**3. Does your manuscript use ONLY data from a publicly available database (eg, SEER, Medicare)?** If "Yes," please specify the database; no additional information is needed. If "No," please complete questions 4-17.	No
**4. Will the data collected for your study be made available to others?** (If "No," enter context for your decision)	Anonymized individual participant data that underlie the results reported in this article will be shared after deidentification to investigators whose proposed data use has been approved by investigators of the JCOG Head and Neck Cancer Study Group identified for that purpose.
**5. List which data are available**	Anonymized individual participant data that underlie the results reported in this articl
**6. Additional information about the data** (Enter "None," if applicable)	None
**7. List how or where the data can be obtained** (e.g., e-mail address, URL, or other repository)	Proposals should be directed to matahara@east.ncc.go.jp.
**8. List beginning and end dates that data will be available** (e.g., MM/DD/YYYY to MM/DD/YYYY)	10/16/2012-04/20/2024
**9. List any supporting documents** (Enter "None," if applicable)	None
**10. Enter additional information about supporting documents** (Enter "None," if applicable)	None

**11. How or where can supporting documents be obtained?** (e.g., e-mail address, URL, or other repository)	None
**12. List beginning and end dates that supporting documents will be available** (e.g., MM/DD/YYYY to MM/DD/YYYY)	None
**13. Indicate to whom data will be available** (Enter "U/K" for unknown; "N/A" for not applicable)	N/A
**14. Indicate for what type of analysis or purpose** (Enter "U/K" for unknown)	U/K
**15. Indicate by what mechanism** (Enter "U/K" for unknown)	U/K
**16. Enter any other restrictions** (Enter "None," if applicable)	None
**17. Enter any additional information** (Enter "None," if applicable)	None

(Optional) If you have included or would like to include a Data Sharing Statement in addition to the above, please provide on the following page.

Data Sharing Statement
